# Magnetic resonance imaging radiomics-based prediction of severe inflammatory response in locally advanced rectal cancer patients after neoadjuvant radiochemotherapy

**DOI:** 10.1007/s00423-024-03416-7

**Published:** 2024-07-17

**Authors:** Li Chen, Wenchao Zhu, Wei Zhang, Engeng Chen, Wei Zhou

**Affiliations:** 1 Department of Colorectal Surgery, School of Medicine, Sir Run Run Shaw Hospital, Zhejiang University, Hangzhou, China; 2 Department of Radiology, School of Medicine, Sir Run Run Shaw Hospital, Zhejiang University, Hangzhou, China

**Keywords:** Radiomics, Magnetic resonance imaging, Rectal cancer, Neoadjuvant therapy

## Abstract

**Purpose:**

To predict severe inflammatory response after neoadjuvant radiochemotherapy in locally advanced rectal cancer (RC) patients using magnetic resonance imaging (MRI) radiomics models.

**Methods:**

This retrospective study included patients who underwent radical surgery for RC cancer after neoadjuvant radiochemotherapy between July 2017 and December 2019 at XXX Hospital. MRI radiomics features were extracted from T2WI images before (pre-nRCT-RF) and after (post-nRCT-RF) neoadjuvant radiochemotherapy, and the variation of radiomics features before and after neoadjuvant radiochemotherapy (delta-RF) were calculated. Eight, eight, and five most relevant features were identified for pre-nRCT-RF, post-nRCT-RF, and delta-RF, respectively.

**Results:**

Eighty-six patients were included and randomized 3:1 to the training and test set (n = 65 and n = 21, respectively). The prediction model based on delta-RF had areas under the curve (AUCs) of 0.80 and 0.85 in the training and test set, respectively. A higher rate of difficult operations was observed in patients with severe inflammation (65.5% vs. 42.9%, P = 0.045).

**Conclusion:**

The prediction model based on MRI delta-RF may be a useful tool for predicting severe inflammatory response after neoadjuvant radiochemotherapy in locally advanced RC patients.

**Supplementary Information:**

The online version contains supplementary material available at 10.1007/s00423-024-03416-7.

## Introduction

Rectal cancer (RC) ranks eighth among cancer incidences worldwide, with 732,210 new cases in 2020 and 339,022 deaths [1]. RC is considered to result from an accumulation of genetic and epigenetic alterations [2]. Many patients present with a locally advanced disease, which carries a 5-year survival rate of 71% [3]. The international standard treatment for locally advanced RC is total mesorectal excision after neoadjuvant radiochemotherapy [4–8]. Still, some tumors might be too large for resection or at risk of partial resection. Neoadjuvant therapy can shrink the tumor, thus improving the resection rate and patient prognosis [9–11]. Indeed, patients with locally advanced RC are difficult to treat with surgery alone, and a multidisciplinary approach is often necessary [8].

Radiochemotherapy can have side effects like inflammation and fibrosis [12, 13]. Chemotherapy induces tumor cell death that can lead to a local anti-tumor inflammatory effect [14–16]. The death of the tumor cells and the inflammatory microenvironment will also lead to the recruitment of myeloid cells that will initiate a kind of wound-healing process [15, 16]. The intensity of the inflammatory and fibrotic responses to radiochemotherapy appears to be associated with treatment efficacy, and a more severe response could indicate a more complete eradication of the tumor cells [15, 16]. At present, it is difficult for clinicians to observe by colonoscopy and traditional imaging whether a pathological complete response (pCR) or the inflammatory response of RC has been achieved after neoadjuvant therapy but before the operation. Nevertheless, evaluating such a response is important to help evaluate the effect of the neoadjuvant therapy. In addition, evaluating the inflammatory response could help determine whether the treatment is effective and formulate the management strategy.

Magnetic resonance imaging (MRI) has played an increasingly important role in diagnosing RC in recent years, often used as the gold standard for preoperative clinical staging of advanced RC. Usually, T2-weighted MRI sequences (T2WI) can clearly show hyper-intensity on the mucosal surface of the rectal and intestinal walls and hypo-intensity on the muscular layer of the intestinal wall. Still, the MRI-based determination of RC is affected by many factors, such as the skill level of the diagnostician, and it is often subjective [17–19]. As a non-invasive and effective means of prediction and recognition, radiomics-assisted diagnosis technology has great potential in cancer research [20, 21]. Recently, radiomics, especially MRI radiomics, has been extended to all aspects of the diagnosis and treatment of CRC, including distinguishing CRC from early cancer and predicting T stage, lymph node metastasis, peripheral nerve invasion, tumor treatment efficacy, microsatellite instability (MSI), and B-RAF status [22, 23]. The radiomics features (RFs) can be quantified to obtain a score, which can be compared pre/post-treatment to quantify the differences after treatment (delta-RF) [24, 25].

Still, presently, severe inflammatory response after neoadjuvant radiochemotherapy can only be confirmed by the histopathological examination of surgically removed specimens. Therefore, this study aimed to establish a prediction model based on MRI radiomics features to detect severe intestinal wall inflammatory response after neoadjuvant radiochemotherapy for locally advanced RC.

## Methods

### Study design and patients

This retrospective study included the patients who underwent radical surgery for RC cancer after neoadjuvant radiochemotherapy between July 2017 and December 2019 at XXX Hospital. This study was approved by the ethics committee of XXX Hospital. The requirement for individual informed consent was waived by the committee.

**Fig. 1:**
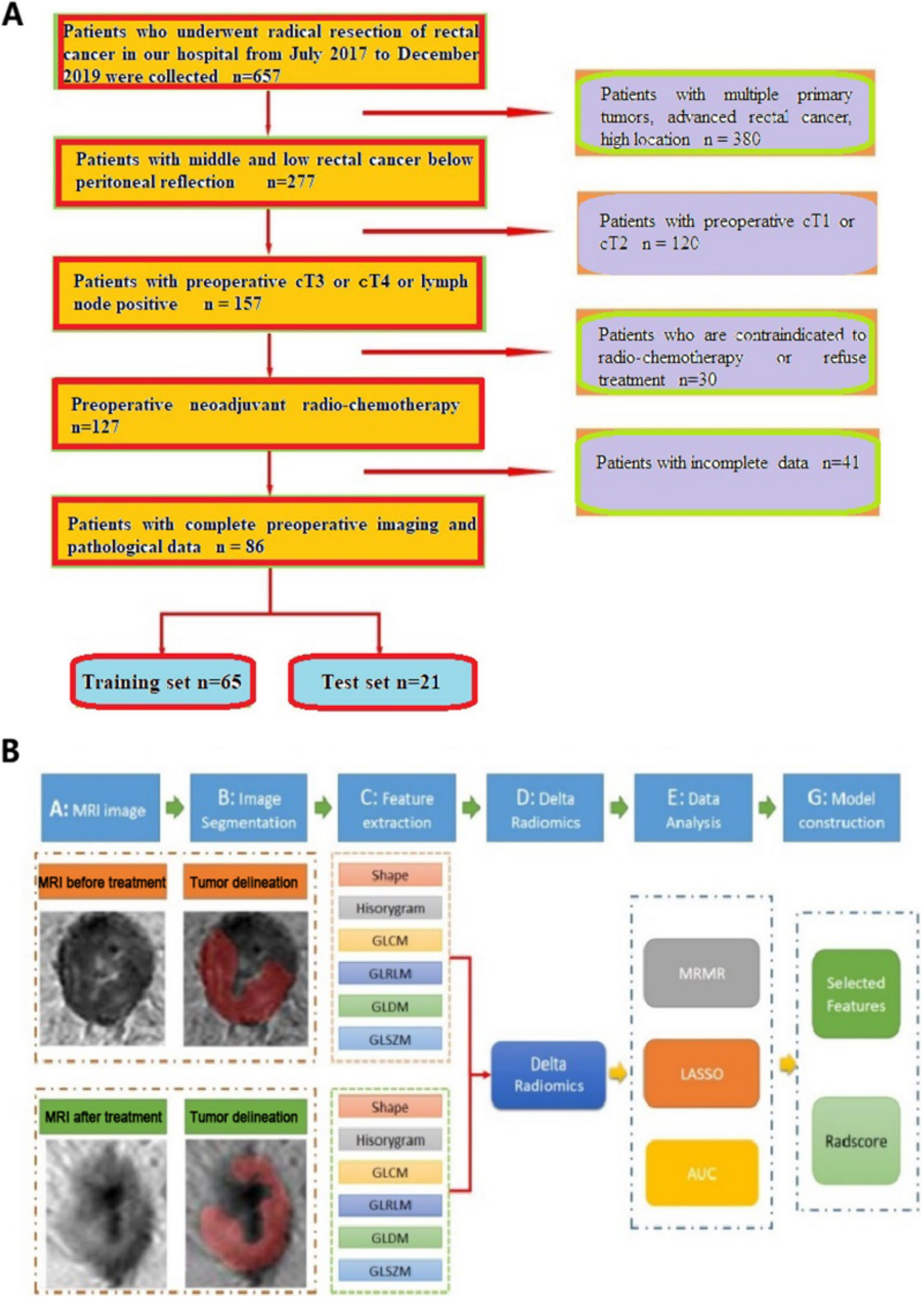
Flowchart of patient enrollment (**A**) and flowchart of the experimental scheme (**B**)

The inclusion criteria were 1) locally advanced rectal adenocarcinoma (preoperative cT3 or cT4 or cN +), 2) underwent standard radical resection of RC after neoadjuvant radiochemotherapy, and 3) underwent pelvic MRI examination before and at the end of neoadjuvant radiochemotherapy. The exclusion criteria were 1) multiple primary tumors, 2) interruption of radiochemotherapy or did not receive radiochemotherapy according to the treatment plan, or 3) incomplete data. The patients were randomized 3:1 to the training and test set **(**Fig. 1**)**.

### Treatment

**Fig. 2:**
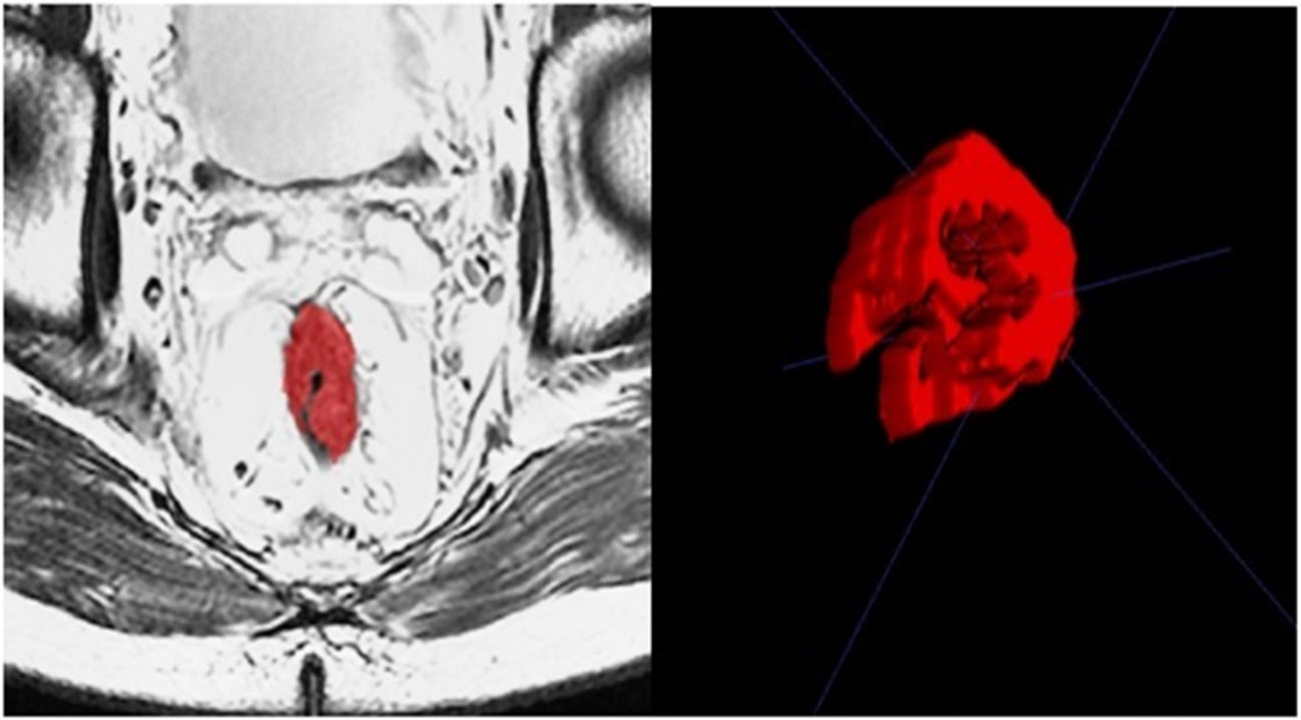
Schematic diagram of rectal tumor delineation completed by ITK-SNAP

The treatment plan and schedule of all patients followed the guidelines of the National Comprehensive Cancer Network (NCCN), which were current when the patients were treated. During the study period, the standard neoadjuvant radiochemotherapy regimen was intensity-modulated radiation therapy (IMRT) at 45–50 Gy in 25 fractions (1.8–2 Gy/fraction), which was completed in 35–42 days, combined with oral capecitabine 825 mg/m^2^, twice a day. Radical resection of rectal cancer was performed by colorectal surgeons with > 10 years of experience 6–9 weeks after neoadjuvant treatment. After surgery, all patients received oxaliplatin 130 mg/m^2^ d1 and capecitabine 1000 mg/m^2^ twice daily d1-14, every 21-day cycle, for 4–5 cycles (Fig. 2).

### Data collection

This study collected the clinical and pathological indexes of the patients at baseline: age, sex, distance from the tumor to the anal margin, pathological differentiation, and TNM stage. All the data were from the medical records. The pathological characteristics and radiomics features were reappraised according to the surgical specimens and MRI imaging. The distance between the tumor and the anal margin was measured on the T2WI images as the distance between the lower edge of the tumor and the anal margin. The preoperative staging was revised according to the 8th edition of the AJCC Guidelines [26]. The patients whose operation time was < 205 min and whose intraoperative blood loss was < 60 ml were classified as routine operations, and the others were classified as difficult operations.

### Pathological evaluation

**Fig. 3:**
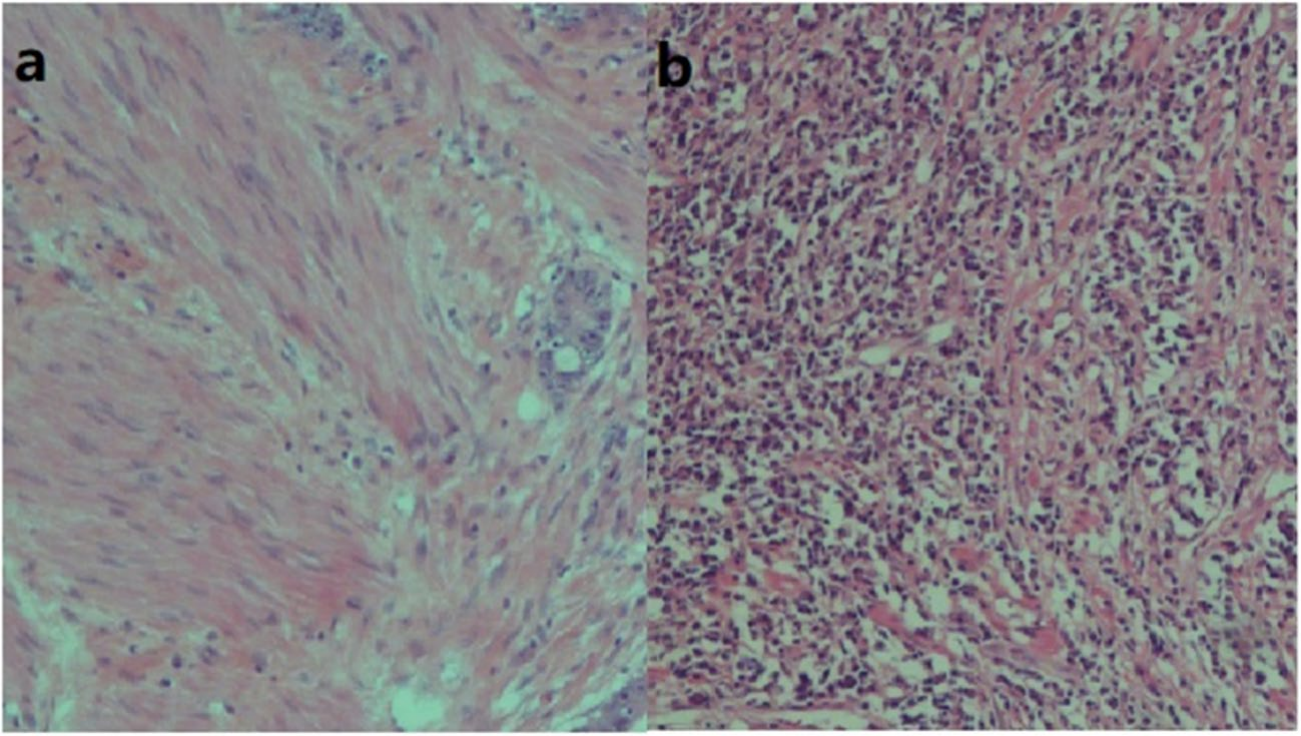
Mild (**a**) and severe (**b**) inflammatory reaction of the rectal wall after neoadjuvant radiochemotherapy (hematoxylin & eosin staining, × 100)

Formalin-fixed paraffin-embedded tissue blocks were sectioned to 5 µm. The sections were stained using routine hematoxylin & eosin staining. The evaluation was performed by a pathologist with > 10 years of experience in diagnosing gastrointestinal tumors. The response of RC to neoadjuvant therapy was mostly shown as diffuse fibrosis with inflammatory cell infiltration. The pathological evaluation criteria referred to the 8th edition of the AJCC Guidelines [26]. The inflammatory response of the rectal wall where the tumor was located was graded according to the infiltration of inflammatory cells: grade 0 (absolutely no inflammatory response), grade 1 (mild inflammatory response), grade 2 (moderate inflammatory response), and grade 3 (severe inflammatory response). Grade 0–1 was considered mild inflammation, and grade 2–3 was severe **(**Fig. 3**)**. The patients were divided into mild and severe inflammation according to the severity of the postoperative pathological inflammation.

### Magnetic resonance imaging examination data

During the study period, the patients routinely underwent a pelvic enhanced MRI before radiochemotherapy, during treatment, and before radical surgery. All MRI examinations were performed using a 3.0-T Siemens magnetic resonance scanner, using an 8-channel phased array coil in the supine position. Conventional MRI sequences included sagittal T2WI, coronal T2WI parallel to the long axis of the lesion, pelvic T1WI, and diffusion-weighted imaging (DWI) with b values of 0 and 800 s/mm^2^, respectively. All MRI data were retrieved from the medical Picture Archiving and Communication System (PACS).

### MR image acquisition parameters

All patients included in this study underwent high-resolution rectal MRI examination using a 3.0-T scanner. All examinations were performed using two devices: the GE MR Signa HDx 3.0 T and the Siemens MR Skyra 3.0 T. Patients fasted for more than 4 h before the MR examination. In addition to the routine contrast-enhanced rectal MR examination, all patients underwent a high-resolution, small field-of-view T2WI without fat suppression. For high-rectal and mid-rectal cancer, the imaging was performed perpendicular to the tumor segment. For low-rectal cancer, the imaging was done perpendicular to the tumor segment and parallel to the coronal plane of the anal canal. The parameters for the GE MR Signa HDx 3.0 T were as follows: 8-channel phased array torso coils; TR/TE = 5000/100 ms; FOV = 16 cm × 16 cm; NSA = 2; slice thickness = 3 mm; slice gap = 0.3 mm; matrix = 352 × 224. The parameters for the Siemens MR Skyra 3.0 T are as follows: 18-channel phased array torso coils; TR/TE = 5000/100 ms; FOV = 16 cm × 16 cm; NSA = 2; slice thickness = 3 mm; slice gap = 0.3 mm; matrix = 320 × 272.

### Tumor delineation

The features were extracted from T2WI and DWI sequences. The imaging examinations were performed on T1WI sequences. The region of interest (ROI) was manually delineated in the MRI images by one radiologist with > 5 years of experience in the diagnosis of gastrointestinal tumors using ITK-SNAP **(**Fig. 2**)**. The ROIs were selected based on clinical experience. Another imaging expert with > 10 years of experience diagnosing digestive tract tumors reviewed and revised the ROIs after discussion with the first radiologist. This study delineated a total of 432 levels of ROI, of which 3.5% were modified, suggesting high consistency. The selection of the ROI included all tumors and the corresponding rectal wall while avoiding blood vessels and intestinal gas. The T2WI images of RC show a slightly high signal before radiochemotherapy, and the ROI was placed in the area of high signal intensity. After radiochemotherapy, if low, mixed, or abnormal rectal wall signals were detected at the original tumor location on T2WI, the ROI was drawn with the abnormal signal area as the outline. If no abnormal or high signal was detected at the original tumor location on T2WI, the original tumor location area was used as the outline during ROI delineation.

### Radiomics feature extraction

The Python 3.0 pyradiomics package was used to extract the RFs before (pre-nRCT-RF) and after (post-nRCT-RF) neoadjuvant radiochemotherapy, including first-order statistics feature (first order), GLCM (grey-level co-occurrence matrix), GLRLM (gray-level run-length matrix), GLSZM (gray-level size zone matrix), and GLDM (gray-level dependence matrix). The obtained radiomics features were standardized, with a total of 960 features. The variations of the radiomics features before and after neoadjuvant radiochemotherapy (delta-RF) were calculated using the following formulas: Delta-RF = (post-nRCT-RF—pre-nRCT-RF) / pre-nRCT-RF.

The redundant and irrelevant features were eliminated in advance using the minimum redundancy maximum relevance (mRMR) method. Using the mRMR algorithm, the top 20 features were selected from the original 960 features. Then, the least absolute shrinkage and selection operator (LASSO) was used to reduce the dimension and select the most valuable features as the input features of the final logistic regression model. The above work was completed using R 3.6.3.

The radscores were calculated for each patient by a linear combination of the selected features and coefficient vectors. The receiver operating characteristics (ROC) curves were drawn, and the areas under curves (AUCs) were calculated.

### Statistical analysis

SPSS 22.0 (IBM, Armonk, NY, USA), Python 3.0, and R 3.6.3 were used for statistical analysis. All continuous data were tested for normal distribution using the Shapiro–Wilk test and conformed to the normal distribution; they were described as means ± standard deviations and analyzed using Student’s t-test. The categorical data were described as n (%) and analyzed using the chi-square test. The Wilcoxon signed-rank test was used to compare the differences in texture parameters before, during, and after neoadjuvant radiochemotherapy. Two-sided P < 0.05 were considered statistically significant.

## Results

### Demographic characteristic

Eighty-six patients were included in this study: 50 (58.1%) males and 36 (41.9%) females. Twenty-eight patients were included in the mild inflammation group, and 58 patients were included in the severe inflammation group; there were no significant differences in demographic and clinical characteristics between the two groups (all P > 0.05) **(**Supplementary Table S1**)**. The patients were randomized 3:1 to the training and test set (n = 65 and n = 21, respectively). There were no significant differences in demographic and clinical characteristics between the mild and severe inflammation groups within each set (all P > 0.05) **(**Table 1**)**.

**Table 1:** Comparison of clinical and pathological data between the mild and severe inflammation groups

Characteristics	Training set, n=65		P	Test set, n=21		P
	Mild inflammation group (n=23)	Severe inflammation group (n=42)		Mild inflammation group (n=5)	Severe inflammation group (n=16)	
Age	60.39±7.71	61.45±7.39	0.593	61.4±5.24	60.13±11.31	0.819
Sex			0.672			0.525
Male	13	26		2	9	
Female	10	16		3	7	
Distance from the anal margin			0.413			0.375
0-5 cm	15	23		3	6	
6-10 cm	8	19		2	10	
Pathological differentiation			0.749			0.920
Poorly differentiated	13	22		3	10	
Medium to high differentiated	10	20		2	6	
T stage before treatment			0.750			0.951
T0	0	0		0	0	
T1	0	0		0	0	
T2	0	1		0	0	
T3	21	37		4	13	
T4	2	4		1	3	
T stage after treatment			0.646			0.229
T0	6	5		1	0	
T1	3	6		2	4	
T2	4	10		0	1	
T3	9	20		2	11	
T4	1	1		0	0	
N stage before treatment			0.597			0.375
N0	1	4		0	0	
N1	9	19		3	6	
N2	13	19		2	10	
N stage after treatment			0.420			0.152
N0	16	33		5	11	
N1	7	9		0	5	
N2	0	0		0	0	

The average operation time was 205 (135–320) min. The intraoperative blood loss was 60 (20–100) ml. A higher rate of difficult operations was observed in patients with severe inflammation (65.5% vs. 42.9%, P = 0.045) **(**Table 2**)**.

**Table 2:** Relationship between inflammatory reaction around tumor and surgical difficulty

Groups	Mild inflammation group (n=28)	Severe inflammation group (n=58)	P
Routine operation	16	20	0.045
Difficult operation	12	38	

### Radiomics feature selection

**Fig. 4:**
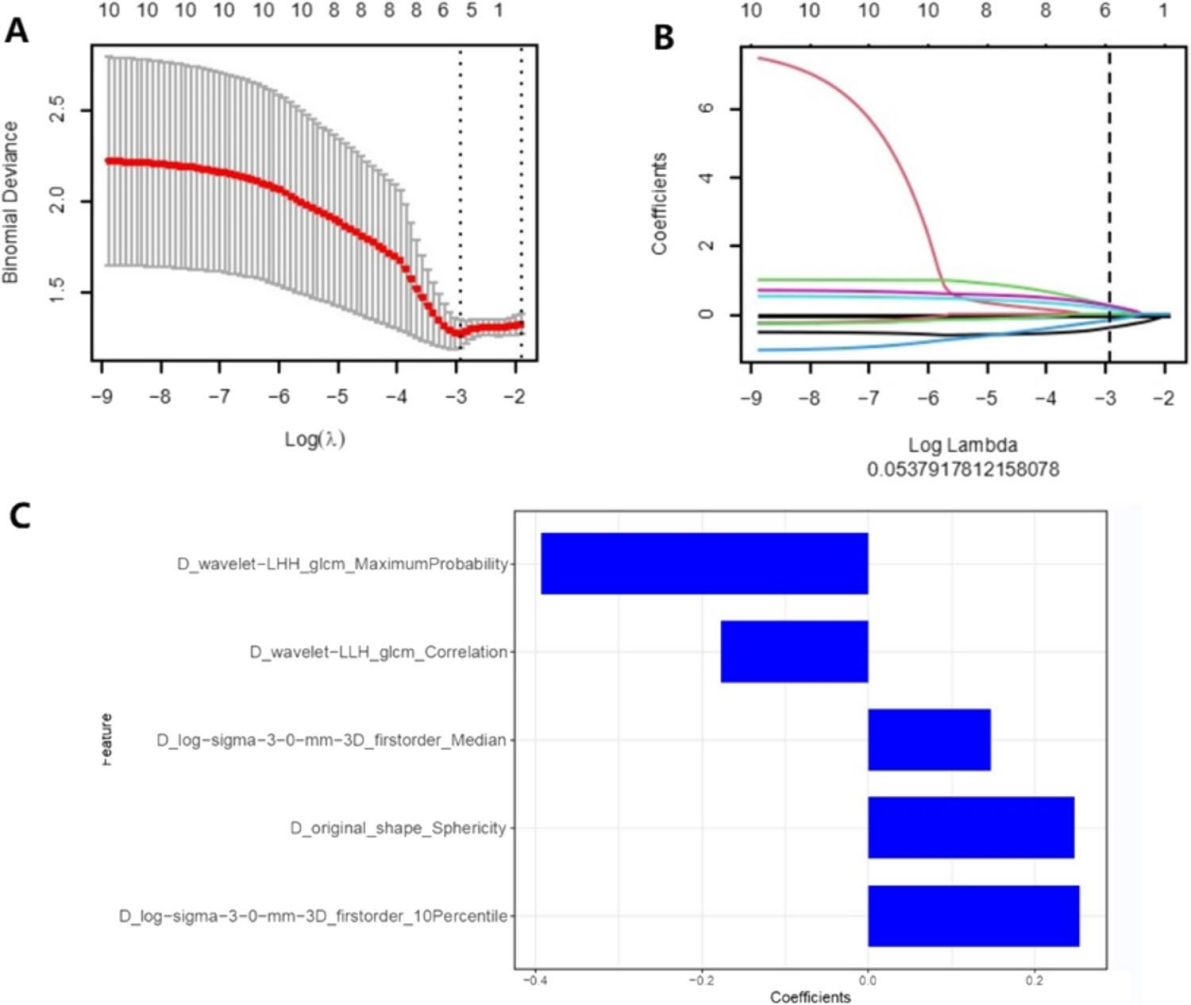
Delta-radiomics selected texture features through the least absolute shrinkage and selection operator (LASSO) binary logistic regression model

A total of 960 radiomics features were obtained after standardized processing. The LASSO binary logistic regression model screened the eight, eight, and five most relevant features to predict the pathological inflammatory response for pre-nRCT-RF, post-nRCT-RF, and delta-RF **(**Fig. 4**)**. The LASSO binary logistic regression model screened the five most relevant features (D_wavelet-LHH_glcm_MaximumProbabilty, D_wavelet-LLH_glcm_Correlation, D_Iog-sigma-3–0-mm-3D_firstorder_Median, D_oniginal_shape_Sphericity, D_log-sigma-3–0-mm-3D_firstorder_10Percentile) to predict the pathological inflammatory response for delta-RF.

### Prediction model for the severe inflammatory response

**Fig. 5:**
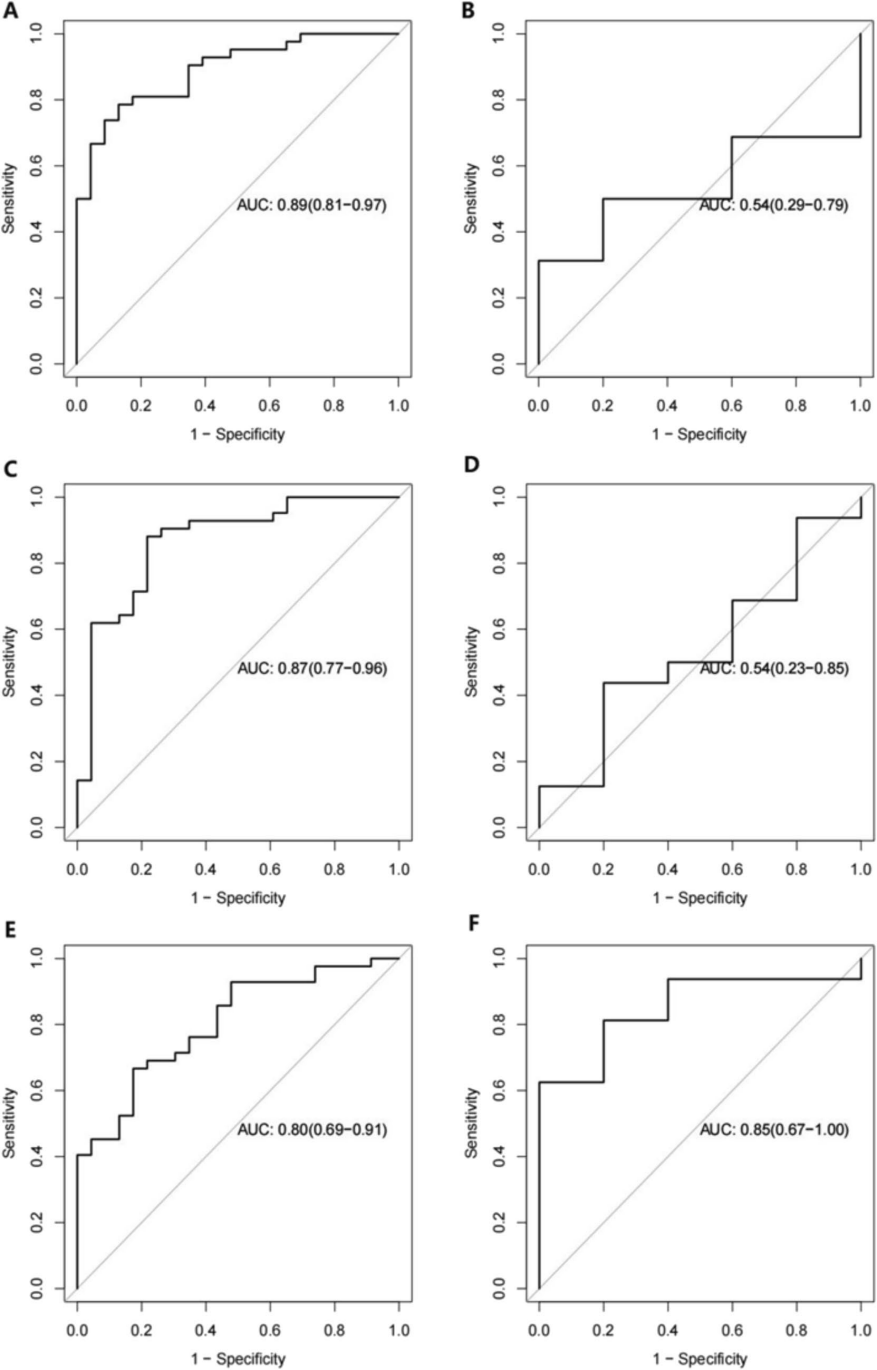
Comparison of receiver operating characteristics (ROC) curves of the training and test set of three models. The radiomics before neoadjuvant radiochemotherapy (pre-nRCT) in the training (**A**) and test (**B**) set. The radiomics before radical surgery (post-nRCT) in the training (**C**) and test (**D**) set. The delta-radiomics in the training (**E**) and test (**F**) set

The pre-nRCT-RF model predicted the pathological inflammatory response with AUC in the training and test set of 0.89 (95%CI: 0.814, 0.967) and 0.54 (95%CI: 0.286, 0.790), respectively **(**Fig. 5A-B**)**. The pre-nRCT-RF model predicted the pathological inflammatory response with sensitivity and specificity in the training set of 0.786 and 0.87, respectively. The pre-nRCT-RF model predicted the pathological inflammatory response with sensitivity and specificity in the test set of 0.616 and 0.313, respectively.

The post-nRCT-RF model predicted the pathological inflammatory response with AUCs in the training set and test set of 0.87 (95%CI: 0.771, 0.964) and 0.54 (95%CI: 0.229, 0.846), respectively **(**Fig. 5C-D**)**. The post-nRCT-RF model predicted the pathological inflammatory response with sensitivity and specificity in the training set of 0.88 and 0.783, respectively. The post-nRCT-RF model predicted the pathological inflammatory response with sensitivity and specificity in the test set of 0.782 and 0.625, respectively.

The delta-RF model predicted the pathological inflammatory response with the AUCs in the training and test set of 0.80 (95%CI: 0.695, 0.910) and 0.85 (95%CI: 0.672, 1.000), respectively **(**Fig. 5E-F**)**. The delta-RF model predicted the pathological inflammatory response with sensitivity and specificity in the training set of 0.667 and 0.826, respectively. The delta-RF model predicted the pathological inflammatory response with sensitivity and specificity in the test set of 0.813 and 0.8, respectively.

## Discussion

There is a lack of radiomics research in predicting the side effects of radiochemotherapy for locally advanced RC. The present study performed a radiomics analysis on the pre-nRCT and post-nRCT MRI images of locally advanced RC. This study developed and evaluated the performance of a delta-RF model, which is based on the two MRI texture parameters to predict the inflammatory response of the intestinal wall after treatment, and the delta-RF model using T2WI best predicted inflammatory response. The results suggest that delta-RF is a potential imaging marker that can help clinicians guide their work and evaluate the side effects of neoadjuvant therapy for RC. Elaborating the most optimal treatment strategy involves gathering as much information about the tumor response as possible, including the inflammatory status of the lesion.

In this study, the pathological scores of inflammation in tissues around locally advanced RC after radiochemotherapy were used to classify the degree of tissue inflammation. The scoring system in this study is referred to as the tumor regression grading system. This system helps evaluate the treatment efficacy of neoadjuvant therapy [27]. According to the CSCO 2022 guidelines, TRG from 0 to 3 corresponds to different tumor regression states [28]. In the present study, the severity of inflammation was not associated with any demographic or clinical variables except with the complexity of the surgery. Therefore, age and sex were unrelated to the inflammatory response, suggesting that the inflammatory response after treatment is independent of those factors. Radiochemotherapy has acute effects on the tissues that are too important to be influenced by sex hormones or age. Although the efficacy and adverse effects of some chemotherapy regimens display differences between males and females, other regimens show similar adverse events; still, little is known about the sex disparities in adverse events to radiochemotherapy [29]. On the other hand, older age is often associated with frailty and lower functional reserves to face the adverse events of chemotherapy [30, 31]. Still, the sample size in the present study was relatively small, which could influence the results.

It has been suggested that the extent of the inflammatory and fibrotic responses of the tumor to radiochemotherapy appears to be associated with treatment efficacy [15, 16]. On the other hand, the inflammatory reaction can also affect the normal tissues around the tumor, which can increase the difficulty of the surgery [32]. Indeed, edema, exudation, and fibrosis can lead to unclear anatomical spaces, easy bleeding, and longer operation times for tissue dissociation, and edema can also result in longer anastomosis-related steps [33]. Therefore, a high tumor response to neoadjuvant radiochemotherapy can lead to a high inflammatory response of the tumor (associated with a good prognosis) and the surrounding tissues, complication surgery (associated with complications, morbidity, and a poorer prognosis). The present study showed a correlation between the severity of inflammation around the tumor and the difficulty of surgery, but there was no data regarding the oncological prognosis; additional studies will have to examine that issue. In addition, some studies suggested that the degree of inflammation in some patients will decrease when the waiting interval after neoadjuvant radiochemotherapy is prolonged [34–38]. Hence, it might suggest that the strategy and timing of surgery might be adjusted according to the inflammatory response. The timing of surgery for rectal cancer patients after radiochemotherapy requires finding a balance between oncology and inflammatory response. The present study was not designed to determine that timing and additional studies are necessary. Hence, even though the oncological impact might be limited, the impact on surgery could be non-negligible.

Delta-RF can capture rich information about the heterogeneous changes that may be discarded by single time point radiomics [39, 40]. Indeed, Wan et al. [39] used delta-radiomics to predict the pCR of RC to neoadjuvant radiochemotherapy. Jeon et al. [41] developed delta-radiomics models to predict local recurrence, distant metastasis, and disease-free survival in patients with RC. Other studies also showed the application of delta-RF in predicting the response to immunotherapy in patients with non-small-cell lung cancer [40] or neoadjuvant chemotherapy in breast cancer [42]. Although single-point radiomics can provide important information about a disease and even present predictive variables for treatment response, it cannot evaluate the response to the treatment. The advantage of delta-radiomics is precisely quantifying the imaging response to therapy. In the present study, delta-RF could predict the inflammatory response of the intestinal wall after treatment. Additional studies are necessary to examine the value of delta-RF and examine long-term outcomes like recurrence, metastasis, and survival.

This study has limitations. First, as a retrospective study, there were no external verifications of the results. It is necessary to conduct prospective studies with a larger sample size to confirm the findings. Second, tumor segmentation and texture feature extraction were only performed at a single layer. Although layer-by-layer segmentation can reflect the overall characteristics of tumors more comprehensively, its practical application in clinical practice is limited to a certain extent because it is time-consuming. Third, this study mainly focused on the texture parameters of T2WI, and it is necessary to study the predictive value of texture analysis using other sequences. Finally, it must be highlighted that radiomics examines hundreds of imaging features that are, most of the time, not even perceptible to the naked eye or with the help of traditional image examination software. Radiomics is often considered a “black box” that provides results based on the extraction and analysis software, but the parameters cannot be related to any apparent pathological or physiological feature [43–45].

In conclusion, a prediction model based on MRI delta-RF might predict severe inflammatory response after neoadjuvant radiochemotherapy in patients with advanced RC. The results might help surgeons better evaluate patients and optimize the operation.

### Supplementary Information

Below is the link to the electronic supplementary material.

Supplementary file1 (DOCX 15 KB)

## Data Availability

The datasets generated and/or analyzed during the current study are not publicly available due to protecting individual patient privacy but are available from the corresponding author upon reasonable request.

## Abbreviations

ADC: Apparent diffusion coefficient
AUC: Area under the curve
cCR: Clinical complete response
CRC: Colorectal cancer
CSCO: Chinese Society of Clinical Oncology
delta-RF: Variation of radiomics features before and after neoadjuvant radiochemotherapy
DWI: Diffusion-weighted imaging
GLCM: Gray-level co-occurrence matrix
GLDM: Gray-level dependence matrix
GLRLM: Gray-level run-length matrix
GLSZM: Gray-level size zone matrix
IMRT: Intensity-modulated radiation therapy
LASSO: Least absolute shrinkage and selection operator
MDT: Multidisciplinary treatment
MRI: Magnetic resonance imaging
MSI: Microsatellite instability
NCCN: National Comprehensive Cancer Network
PACS: Picture archiving and communication system,
pCR: Pathological complete response
PET: Positron emission tomography
pre-nRCT-RF: Radiomics features before neoadjuvant radiochemotherapy
post-nRCT-RF: Radiomics features after neoadjuvant radiochemotherapy
RC: Rectal cancer
RCT: Radiochemotherapy
RF: Radiomics features
ROC: Receiver operating characteristics
ROI: Region of interest
TME: Total mesorectal excision
TRG: Tumor regression grade
TR: Repetition time
TE: Echo time
FOV: Field of view
NSA: Number of signal average
