# Supplementary material for: Magnetic resonance imaging radiomics-based prediction of severe inflammatory response in locally advanced rectal cancer patients after neoadjuvant radiochemotherapy
Source: Langenbecks Arch Surg. 2024 Jul 17;409(1):218. doi: 10.1007/s00423-024-03416-7 (PMC11255083; doi:10.1007/s00423-024-03416-7)
Supplement: Supplementary file 1 — Supplementary file1 (DOCX 15 KB) [file 423_2024_3416_MOESM1_ESM.docx]

**Supplementary Table S1.** Comparison of clinical information and pathology between mild inflammation group and severe inflammation group

| Characteristics | Mild inflammation (n=28) | Severe inflammation (n=58) | P |
| --- | --- | --- | --- |
| Age | 60.57±7.34 | 61.09±8.67 | 0.790 |
| Sex |  |  | 0.551 |
| Male | 15 | 35 |  |
| Female | 13 | 23 |  |
| Distance from the anal margin |  |  | 0.212 |
| 0-5 cm | 18 | 29 |  |
| 6-10 cm | 10 | 29 |  |
| T stage before treatment |  |  | 0.766 |
| T0 | 0 | 0 |  |
| T1 | 0 | 0 |  |
| T2 | 0 | 1 |  |
| T3 | 25 | 50 |  |
| T4 | 3 | 7 |  |
| T stage after treatment |  |  | 0.295 |
| T0 | 7 | 5 |  |
| T1 | 5 | 10 |  |
| T2 | 4 | 11 |  |
| T3 | 11 | 31 |  |
| T4 | 1 | 1 |  |
| N stage before treatment |  |  | 0.816 |
| N0 | 1 | 4 |  |
| N1 | 12 | 25 |  |
| N2 | 15 | 29 |  |
| N stage after treatment |  |  | 0.931 |
| N0 | 21 | 44 |  |
| N1 | 7 | 14 |  |
| N2 | 0 | 0 |  |

pCR: pathological complete response.
